# Toward Improvements in Pressure Measurements for Near Free-Field Blast Experiments

**DOI:** 10.3390/s23125635

**Published:** 2023-06-16

**Authors:** Maylis Lavayssière, Alexandre Lefrançois, Bernard Crabos, Marc Genetier, Maxime Daudy, Sacha Comte, Alan Dufourmentel, Bruno Salsac, Frédéric Sol, Pascal Verdier, Patrick Pons

**Affiliations:** 1 Commissariat à l’Energie Atomique et aux Energies Alternatives (CEA), Direction des Applications Militaires (DAM), 46500 Gramat, France; alexandre.lefrancois@cea.fr (A.L.); marc.genetier@cea.fr (M.G.); sacha.comte@cea.fr (S.C.); alan.dufourmentel@cea.fr (A.D.); bruno.salsac@cea.fr (B.S.); frederic.sol@cea.fr (F.S.); pascal.verdier@cea.fr (P.V.); 2 Laboratoire d’Analyse et d’Architecture des Systèmes (LAAS-CNRS), Centre National de la Recherche Scientifique (CNRS), Institut National Polytechnique de Toulouse (INPT), Université de Toulouse, 7 Avenue du Colonel Roche, 31031 Toulouse, France; ppons@laas.fr

**Keywords:** pressure sensors, dynamic calibration, transfer function, metrology, shock tube, deconvolution, blast experiment, near-field experimentation, close-in detonation

## Abstract

This paper proposes two ways to improve pressure measurement in air-blast experimentations, mostly for close-in detonations defined by a small-scaled distance below 0.4 m.kg^−1/3^. Firstly, a new kind of custom-made pressure probe sensor is presented. The transducer is a piezoelectric commercial, but the tip material has been modified. The dynamic response of this prototype is established in terms of time and frequency responses, both in a laboratory environment, on a shock tube, and in free-field experiments. The experimental results show that the modified probe can meet the measurement requirements of high-frequency pressure signals. Secondly, this paper presents the initial results of a deconvolution method, using the pencil probe transfer function determination with a shock tube. We demonstrate the method on experimental results and draw conclusions and prospects.

## 1. Introduction

In free-field experiments, when an explosive detonates, a shock wave is propagated through the surrounding air. This blast wave is characterized by its overpressure peak value at the shock front, before decreasing rapidly. This initial positive phase is then followed by a negative phase where the pressure falls below the ambient pressure due to the changing velocity direction of the detonation product gases. The pencil probe geometry has been designed to measure pressure changes in the blast free-field environment, minimizing the distortion created by the measuring device, since it is placed into the space where the blast wave is propagating.

In the experimental field, the devices, for which the first transducers were developed in the 1950s and 1960s [1], are widely used to determine the blast wave characteristics, but the pressure-time history signals shown are scarce. Most often, papers relying on such experiments only deal with some parameters determined from the signals, such as maximum overpressure peak, or positive phase impulsion, under the form of a synthesis of results, but rarely show the temporal signals from which the data have been extracted. Lukić et al. show a statistical analysis of two blast wave parameters, maximum overpressure and pressure-time diagram decay coefficient, based on 21 field tests of the cylindrical 100 g TNT charge free-air detonation, using four quartz free-field ICP^®^ blast pressure pencil probes to measure the incident overpressure [2]. Silver, from the U.S. Army Aberdeen Test Center, presents a methodology for the collection of specific blast overpressure data with pencil probes that are used to develop and analyze the effectiveness of competing cannon designs, to refine and validate Computational Fluid Dynamics models of large-caliber muzzle blasts [3]. Showalter provides an overview of how pencil probes are used in blast experiments conducted by the Explosive Effects Branch of the US Army Combat Capabilities Development Command. Key processes and techniques are explained, along with selection methods for choosing the appropriate probe for the pressure event [4]. Dvořák et al. present another measurement setup to improve the pencil probe measurement, involving different fixation of the probe, during field tests in a military training area [5]. The Norwegian Defense Research Establishment has led an investigation into pencil probe PCB Piezotronics 137A23 misalignment between +10° or −10° that can lead to an overestimation of the peak overpressure of less than 13% in the case of small charges, but that can reach 30% for larger charges [6]. Draganić et al. provide an overview of the devices for pressure measurement, along with a comparison of experimental methods is well performed by [7].

Air-blast parameters are used for designing structural elements to resist blast-induced loads. They include incident and normally reflected peak overpressures and impulses, typically estimated for protective designs using charts developed by Kingery and Bulmash for medium and large-scale distances [8]. However, the graphs underpredict these parameters for small-scaled distances, less than 0.4 m.kg^−1/3^, i.e., close-in detonations [9,10]. The growing need to measure dynamic pressure close to the charge has led to the evaluation of the commercial pencil probe behavior in such a harsh environment.

Hence, reliable overpressure measurements are of prime importance. Bean identified six parameters for the characterization of pressure transducers used in time-resolved measurements: gain, phase lag, resonant frequency, damping ratio, rise time, and overshoot [11]. The blast pressure transducers must be calibrated dynamically, with aperiodic calibrators such as shock tubes. They can generate pressure steps with a fast rise time and a stable amplitude, allowing the study of transducer response in both high and low-frequency ranges [12,13]. Zahnd et al. characterized the pencil probes of two manufacturers on a shock tube to evaluate their performances and the signal quality reachable, but only in the temporal domain [14]. Under laboratory conditions, a shock tube can also be of interest to determine the transfer function according to frequency. We first present the results of the transfer function determination of pencil probe sensors with a shock tube.

This paper focuses on two ways to improve the quality of overpressure measurements: either mechanically, by optimizing the pencil probes, or numerically, with the deconvolution of the temporal signals by the transfer function of the measurement chain. A series of tests were conducted on commercial pencil probes to estimate their limitations, and a new kind of optimized pencil probe was proposed. The evaluation of these mechanical improvements is based on comparing their transfer function, determined from a shock tube in the laboratory. To go further in the improvements of pressure measurement, this transfer function was also used to develop a signal processing method that can be applied to correct output signals from the bandwidth limitations of the measurement system. This work highlights several results determining the advantages of such new probes and paves the way for improving the use of experimental measurements.

The structure of this paper is as follows: Section 2 presents the materials used and the configuration of experiments, setting out the process arbitrarily chosen to evaluate the transfer function of the measurement chain. In Section 3, we present the transfer function of a standard commercial measurement chain and compare it to an optimized pencil probe to enhance the system bandwidth. We then present the methodology developed to increase the information obtained from pressure measurements observed during an air-blast experiment, by deconvoluting the measurement with its transfer function determined in the laboratory.

Finally, in the last section, we discuss the methodology, then draw conclusions, presenting the limitations of such a method and the improvements planned in the deconvolution process.

## 2. Materials and Methods

### 2.1. The Measurement Chain

To characterize a blast-pressure event, two types of pressure transducers for two kinds of measurements can be used:

- Side-on transducers record incident pressure change from the blast source in a free-air blast experiment. Their design, shaped on a pencil probe, minimize interference with the shock wave and the flow behind the shock front. The device’s body is thin, and the tapered probe tip is pointed upstream into the propagating blast wave. Hence, the blast wave should propagate parallel to the longitudinal axis of the pencil probe. This configuration is the one on which this paper focuses;
- Reflected-pressure transducers measure pressures reflected at normal or oblique angles of incidence from a rigid surface, for example, on a wall, where the shock wave reflects. This configuration will not be discussed in this paper.

**Figure 1:**
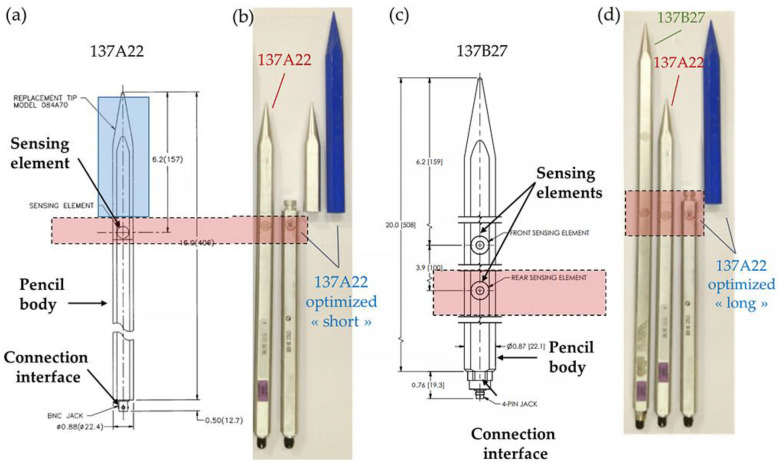
(**a**) Drawing of a PCB Piezotronic 137A22 with the replacement tip zone colored in blue. Dimensional values are given in inch and (mm) [15]. (**b**) Photograph of the 137A22 probes tested in this paper with a short blue plastic tip. (**c**) Drawing of a PCB Piezotronic 137B27. Dimensional values are given in inch and (mm) [15]. (**d**) Photograph of the 137B27 probe, showing its greater length regarding the 137A22 probe, and modified 137A22 with long blue plastic tip. In both sketches and photographs, the red zone shows the sensing element of interest in this paper, on the unique transducer element of the 137A22 probe and the rear element of the 137B27 probe.

This paper focuses on two families of Integrated Circuit PiezoElectric (ICP^®^) pencil probes, from PCB Piezotronics manufacturers, some of whose electrical specifications are summarized in Table 1:

- 137A, with a single ICP^®^ sensing element with a resonant frequency of approximately 500 kHz, where the tip material can be changed (137A22 reference), presented in Figure 1a;
- 137B, consisting of two ICP^®^ sensing elements, presenting a lower resonant frequency of approximately 400 kHz. This reference is presented in Figure 1c. The 137B27 and the 137B28 references have the same features, only the measurement range varies: respectively 35 bar for the B27 and 70 bar for the B28.

These probes are made of three parts, shown in Figure 1a,c: a body, the sensing element(s), and a connection interface. The features of these common probes are similar to most other commercially available sensors. A sensing element is determined by the two rings on the flat side of the body: the transducer (center of the inner ring) consists of a stable quartz piezoelectric element in an Invar (nickel-iron alloy) housing (outer ring). The single sensor for the 137A22 probe, or the front sensor for the 137B28 probe, is located 157 mm from the probe’s tip, such that any turbulence or shock caused by the tip will be dissipated before it reaches the transducer.

The rear of the pencil probe body houses the connection interface that can be composed of two types of connectors:

- a low-noise 10–32 coaxial Jack connector (for the 137B27 probe);
- a Bayonet Neill–Concelman (BNC) connector (for the 137A22 probe).

As will be discussed in this paper, the signals measured by these off-the-shelf probes can be altered by the structure of the probe itself. We introduce an optimized pencil probe. The principle is to replace the metallic tip of a commercial 137A22 pencil pressure probe shown in the blue square of Figure 1a, with a 3D printed plastic one, as shown in Figure 1b,d. Two kinds of plastic tips are used with the same 137A22 body:

- Free-field experiments were conducted with a 137A22 pencil mounted with a “long” tip to adjust the length to the 137B27 commercial one, as shown in the photograph of Figure 1d. The sensing element is always at the same distance from the rear of the probe since this device part is not modified (in the case of the 137B27, it corresponds to the rear sensor);
- An experiment has also been conducted in the laboratory using a 137A22 pencil mounted with a “short” tip, as shown in the photograph of Figure 1b, to adjust the length of the 137A22 commercial pencil probe. The aim is to compare the transfer function of both optimized pencil probes with short or long tips.

Note that the initial transducer in its Invar housing from the commercial probe is still used, without any modification.

The measurement chain configurations for all the sensors presented in this paper are summarized in Table 1.

The aim of changing material is to break the acoustic waves that propagate within the pencil probe when the shock wave impacts the tip. The velocity of the acoustic wave being higher in metal (typically 6320 m/s for aluminum which is the material body of the pencil probe) than the shock wave in the air, it can perturb the pressure transducer, forcing it to resonate, hence inducing noise and parasites on the base level of the signal, and decreasing the arrival time determination of the shock wave on the transducer. Combining both plastic materials for the tip (typically presenting a lower acoustic wave velocity, around 1230 m/s in acrylic, for example) and metal for the body where the transducers are located, limits the acoustic wave velocity and creates an impedance rupture that slows these acoustic waves at the transition, improving the signal to noise ratio. This paper outlines the experimental validation of such a setup.

**Figure 2:**
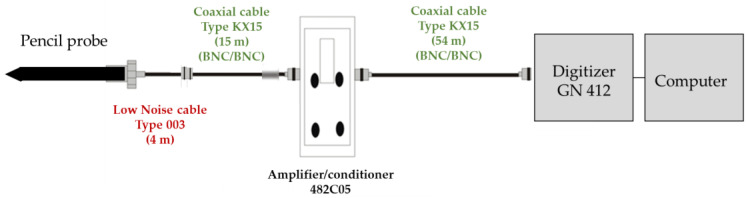
Schematic representation of a measurement chain with cable lengths used in blast free-field campaign.

Outside the transducer itself, the other constituents of the measurement chain must be considered: an amplifier-conditioner, and a transient recorder, made of a digitizer system and a computer to store the data, all connected by cables, as schematically represented in Figure 2.

The conditioner is a PCB Piezotronics 482C05 model, with a voltage gain of 1:1 and a low impedance output of 50 Ω. Its operating principle is related to a voltage amplifier used for decoupling input and output [16]. The output voltage is digitalized on 14 bits resolution recorded at a rate of 20 MSamples.s^−1^ by an HBK GN 412 high-speed input board with a GEN 7t Genesis platform. The input impedance is 1 MΩ.

The main element that degrades the bandwidth of the dynamic measurement system is the pressure sensor itself, the conditioner behaving like an analog low-pass filter with a 1 MHz high cutoff frequency at −3 dB, and the acquisition board presenting an analog bandwidth with a flat response at −3 dB up to 25 MHz. Following the “rule of thumb” given by the manufacturer ENDEVCO [17], the transducer resonance frequency leads to a lower flat frequency range limited to 1/5 of the resonant frequency, hence 80 kHz and 100 kHz for the PCB Piezotronics transducers considered in this paper, respectively 137A and 138B series.

Two types of cables are used in the measurement chain:

- a low-noise coaxial cable, model 003, is used between the sensor and the conditioner. Connected to the sensor, its characteristic impedance is 50 Ω, and its linear capacitance is 99 pF/m;
- a standard KX15 cable is added between the low-noise coaxial cable and the conditioner/amplifier, only for free-field experiments, and is also used between the conditioner and the digitizer. Its characteristic impedance is 50 Ω, and its linear capacitance is 96 pF/m.

The lengths are different between the laboratory and the free-field experiments, as summarized in Table 2.

Considering the cable length before the conditioner: assuming the worst-case scenario where the output impedance value of the pencil probe is 100 Ω, and the conditioner impedance infinite, if we choose a cutoff frequency at −3 dB of 500 kHz (corresponding to the highest resonant frequency of the pressure transducer used in this paper) this implies the use of a cable of 32 m maximum length. The cable length used in the laboratory is well below this value, which means that we can neglect its influence regarding the bandwidth of the whole measurement chain during the characterization of a shock tube.

However, in the free-field experiments, the 4 m low-noise coaxial 003 and the 15 m KX15 coaxial cables are connected in series, resulting in the combined capacitance of a 19 m cable between the probe and the conditioner of 7.38 µF and an impedance of 50 Ω. This length is not negligible: a wave that propagates within the cable will be reflected (due to the deadapted line impedance regarding the source). Considering a propagation velocity of 5 ns/m, the reflection will be total after a duration of 190 ns: it will be impossible to measure transient signals lower than 1 µs without degrading the signal temporally, thus degrading the input response.

Considering the cable components of the measurement chain used at the experimental site, a spectral analysis was performed to characterize its loss. All cables were connected in series, and only the 485C02 conditioner was removed. An Agilent E5061B vector network analyzer in “Full 2 Ports” configuration was used to determine the four S parameters in the frequency range 100 Hz–1 GHz in forward (S_21_ and S_11_) and reverse (S_12_ and S_22_) directions. It considers all the error factors occurring for a measurement on a 2-ports network.

**Figure 3:**
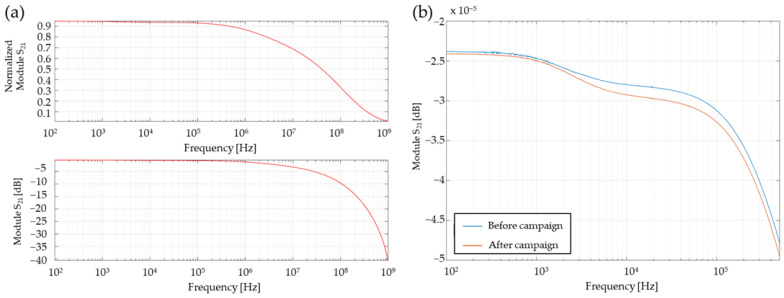
S_21_ parameter module determined by a vector network analyzer in “Full 2 Ports” configuration to characterize the loss of the coaxial measurement chain for an input impedance of (**a**) 50 Ω (experimental data) and (**b**) 1 MΩ (deduced after (**a**) measurements), in blue before the beginning of the campaign and in orange, at the end.

The analyzer characteristic impedance is 50 Ω: Figure 3a shows the S_21_ parameter of the whole coaxial length (“Full 2 Ports”), corresponding to the response of the transmission line when it is loaded with 50 Ω and attacked by a source of internal impedance 50 Ω (corresponding to the analyzer configuration impedance).

The loss evaluation of the experimental chain, corresponding to an input of impedance 1 MΩ due to the Genesis digitizer, was deduced from a voltage divider calculation regarding the measurements obtained for 50 Ω. The S_21_ parameter response spectrum, which corresponds to the scalar linear gain, presented in Figure 3b, shows that there is no influence of this whole cable chain in the bandwidth considered (100 Hz–500 kHz) since the S_21_ module is higher than −5 × 10^−5^ dB. The transmission attenuation of the line at −3 dB is approximately 8.5 MHz.

Note that the graph in Figure 3b shows two spectral responses regarding the input of impedance 1 MΩ for the same measurement chain, before (blue) and after (orange) the free-field detonation campaign: the maximum difference on the module is less than 0.25 × 10^−5^ dB, showing no effect of cables aging due to blast effects on the signal transmission within the bandwidth of interest. These results show that the influence of the cables on the bandwidth of the measurement chain is negligible within the range of frequencies below 500 kHz. The transfer function determination of the measurement chain is mostly governed by the transducer element, the cable used in the laboratory can be taken shorter without influence.

### 2.2. Free-Field Experimental Set-Up

The sensors characterized in this study address pyrotechnic applications and have been used in free-field experiments to measure the maximum overpressure peak resulting from the detonation of a high explosive charge. A campaign was led with two octogen (HMX) based compositions, testing two weights. Some pressure measurements characterizing its effects will be analyzed in this study. Table 3 provides information regarding the HMX weight ratio concerning the polymer binder of these two compositions.

In this campaign, bare charges are studied, and the absence of metallic casing that could create fragmentation helps avoid the risk of impact on and damage to the pencil probes.

All the pencil probes were freshly calibrated by the manufacturer prior to both laboratory and experimental free-field detonation tests. A protective layer was installed over the sensing part of the sensor to thermally insulate the diaphragm from the effect of the fast thermal convection and the electromagnetic radiation (especially in the infrared spectrum) generated during the fireball expansion.

**Figure 4:**
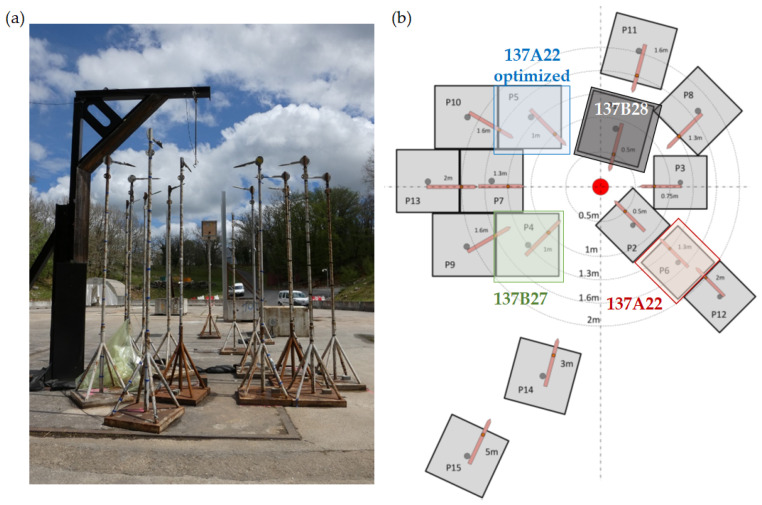
(**a**) Experimental free-field scene. (**b**) Schematic representation of free-field blast sensor installation: the positions of the pencil probes around the charge (red circle) are marked. The identification of the four probes of interest, whose details are given in Table 4, is marked by colored squares.

The distance between the explosive source and the sensors is important to evaluate the change in the maximum overpressure peak relative to the distance from the center of the source. The charge is suspended at a height of 3.5 m from the ground on the black L-shaped bracket in order to avoid ground reflection during the initial steps of shock wave propagation. It is surrounded by an array of blast pencil probes mounted on 3.5 m posts. The locations are sufficiently varied to study the pressure measurements, with varying distances and azimuths. These probes, whose tips are oriented towards the center of the charge, are positioned at several well-known distances from it, as visible in Figure 4a. They should not interfere with each other. Each pencil probe was aligned towards the charge using a laser pointer to limit misalignment and reduce the angle between the direction of propagation of the incoming blast wave and the longitudinal symmetry axis of the pencil probe [6]. Figure 4b shows the sensor positions: they are presented schematically in brown with their support in grey, around the charge represented by the red circle.

In this paper, we focus mostly on three pencil probe sensors surrounded by colored squares in Figure 4b in red, blue, and green, whose configuration is recorded in Table 1 and Table 4. We focus on the measurement improvements given by our optimized 137A22 probe consisting of a plastic tip, called “137A22 optimized”. Their transfer function and behavior are compared in Section 3.1. Section 3.2 focuses on the measurement performed by a fourth pencil probe 137B28 sensor, presented in the grey square of Figure 4b.

The shock wave from an explosion is governed by the distance from the center of the detonation, and the nature and size of the charge. We correlate the effect of the different charges by using the scaled distance *Z* presented in Formula (1):

(1) $$Z=\frac{R}{W^{1/3}}$$

where *R* is the distance between the transducer and the center of the charge, and *W* is the mass of the charge.

Table 5 shows that the free-field experiments conducted in this campaign tend to reach increasingly short-scaled distances.

**Figure 5:**
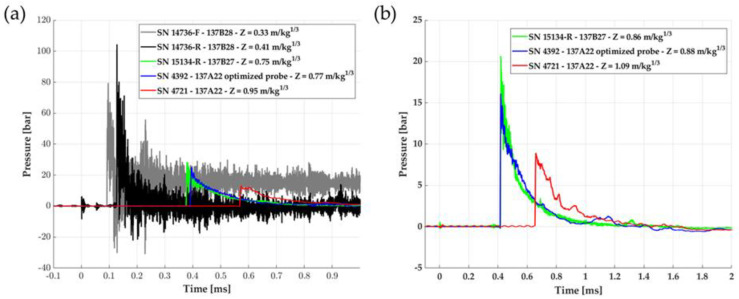
(**a**) Example of raw pressure measurement for 2.459 kg of composition 2 on pencil probe sensors at different Z ranges. (**b**) Example of raw pressure measurement for 1.639 kg of composition 1 on pencil probe sensors at different Z ranges. The pressure measurements by the three sensors of interest in this paper are visible. The signal’s shape is a Friedlander-Type, as expected from a dynamic measurement of blast pressure.

Figure 5a presents an example of the raw pressure measurements resulting from a free-field detonation for 2.459 kg of composition 2: the smaller the scaled distance, the noisier the pressure signals, and the more difficult the determination of the maximum overpressure peak. The gray and black signals correspond to the 127B28 pencil probe the nearest to the charge, respectively 0.45 m for the front transducer (grey) and 0.55 m (black), corresponding to scaled distances respectively of 0.33 m.kg^−1/3^ and 0.41 m.kg^−1/3^. The need to limit the noise is obvious and is of prime importance to improve the data interpretation. The colored signals, coming from pencil probes located farther from the charge, for Z > 0.4 m.kg^−1/3^ are not so noisy. Since the measurements are cleaner at these distances, it explains our choice to test the optimized pencil probe first around a distance of approximately 1 m from the charge to better evaluate the impact of our mechanical modification.

Figure 5b presents an example of the raw pressure measurements resulting from a free-field detonation for 1.639 kg of composition 1 with only the pressure measurements that will be of interest to evaluate the optimized design solution. The 137A22 optimized plastic tip (blue) and the 137B27 (green) see the shock wave almost simultaneously since they are positioned at the same distance from the charge. The 137A22 commercial pencil probe (red) sees the shock wave 0.25 ms after the time arrival detection of the two other sensors since it is located 0.26 m further from the center of the charge.

As we can see from Figure 5, the pressure measurement in real time during these air blasts is very challenging due to the abrupt variation of pressure from the atmospheric pressure to the peak overpressure at the shock front ΔP_max_ (between a few bars and several tens of bars) with a very short rise time t_m_ (<10 ns). The blast wave shows a pressure drop when the overpressure peak is reached. The growing need for dynamic measurements implies correcting the measured signals from the effects of the complete measurement chain bandwidth limitations. We can mention as major drawbacks for air blast experiments: the delay due to both response and rise times, and an overestimation of the input signal maximum peak since this latter can be excited over its bandwidth, leading to a ringing on the signal as can be seen in Figure 5a [12,18].

To characterize such sensors, for a similar measurement chain (same amplifier and same digitizer, but we have seen previously that it was not necessary to consider the same cable length), the frequency-dependence behavior given by the frequency response function must be qualified. The shock tube provides time-domain calibration where gain and phase are identified by the output ratio to the input signals’ Fourier transforms. To compare pencil probe response in the frequency domain, their transfer function will be of use. The next section presents this parameter determination.

### 2.3. The Laboratory Shock-Tube Set-Up for the Transfer Function Determination

**Figure 6:**
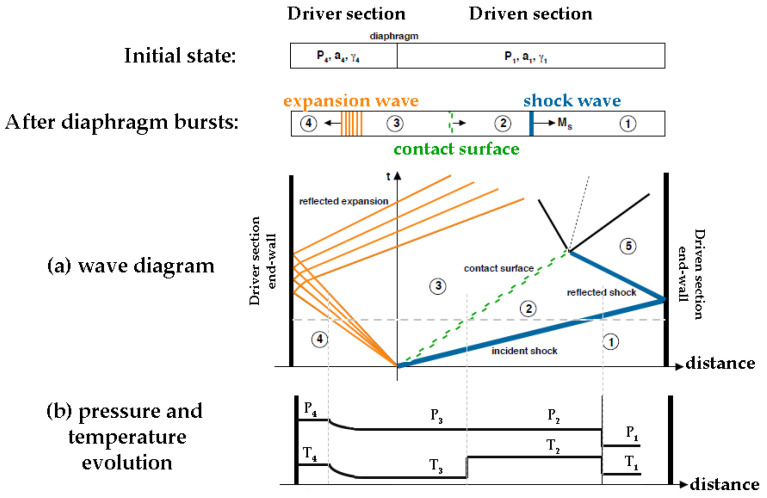
The shock tube experiment in the initial state and after the diaphragm burst: (**a**) wave diagram and (**b**) pressure and temperature evolution with respect to time and distance within the tube. The circled numbers show the different states of the gases defined in the shock tube theory.

In a two-section shock tube, the driver and the driven sections are filled with gases at different initial conditions of pressure, temperature, and adiabatic index, respectively P_4_, T_4_, γ_4_ for the driver section and P_1_, T_1_, γ_1_ for the driven one, as shown on Figure 6. They are separated by a diaphragm. The pressure in the driver section is increased until the diaphragm bursts. Its rupture creates two waves: a shock wave propagating at a pressure P_2_ within the driven section, and an expansion wave that goes into the driver section at a pressure P_3_. The moving boundary between the shock-processed gas and the expanded gas is called the contact surface, represented in the green dash-line in the wave diagram of Figure 6. It moves more slowly than the shock wave behind the shock front, leading to the preservation of both pressure and velocity through the interface, whereas the temperature and density undergo a discontinuity. The incident shock wave, represented by a blue line, travels in the driven section of the tube until it reflects on the end wall to reach the pressure P_5_ and comes back into the gas compressed and heated by the incident shock wave.

Two kinds of pressure steps can be encountered by a pressure transducer depending on its localization:

- When the shock wave passes in the driven section, a sensor located on the tube wall, or on a pencil probe, sees a pressure step of amplitude P_2_–P_1_ sweeping its active surface. P_2_ is also referred to as the incident pressure;
- When the shock wave arrives at the end of the driven section, a sensor on it sees a pressure step of amplitude P_5_–P_1_. This measured pressure will remain stable at P_5_ until the arrival of the contact surface. P_5_ is also called the reflected pressure.

The CEA–Gramat Metrology Laboratory uses a metrological shock tube 0.11 m in diameter and 2.7 m in length [19]. The inner diameter of the tube is 110 mm for an outer diameter of 132 mm, leading to a driver section volume of 8.5 L and a driven section volume of 17 L. The driver section is filled with nitrogen gas, to reach Mach number between 1 and 2. The driven section is left at atmospheric pressure. The diaphragm separating the two sections is a standard nickel rupture disc that provides a full-opening design responding to overpressure within milliseconds. It is calibrated to burst at around ± 10% of the defined pressure. Two side-on pressure transducers located in the wall of the driven section and separated by a well-known distance of 0.5 m determine the shock wave velocity by chronometry.

For a pencil probe positioned inside the driven section, a dedicated end wall with a seal ring is used: it maintains the rear of the pencil probe body centered within the driven tube and guarantees airtightness in the shock tube. The transfer function determination for this kind of sensor is given regarding the first pressure step P_2_, i.e., the incident pressure.

We considered our shock tube generating pressure steps with a rise-time of fewer than 10 ns [20,21]; hence, it can excite all the frequency range that characterize the frequency response of the dynamic pressure sensors in side-on configurations.

### 2.4. Transfer Function Determination

We consider a measurement chain that can be modeled as a Linear Time-Invariant (LTI) system respecting three major properties:

- Linearity: given a linear combination of inputs, the LTI system will produce the same linear combination of the corresponding outputs;
- Time-dependency: the LTI system will always give the same output (up to timing) to a certain input, irrespective of when the input was applied;
- Causality: the LTI system depends only on the present and past input values, not on future inputs.

**Figure 7:**
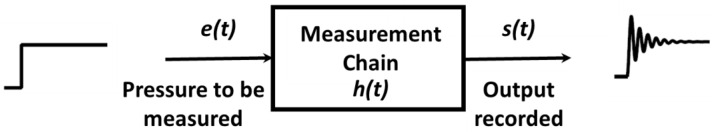
Schematic principle of the influence of the measurement chain transfer function *h*(*t*) on a signal *e*(*t*) to be measured, leading to the recording of the output *s*(*t*).

The dynamic measurement chain can be illustrated (Figure 7): the output recorded is biased by the measurement chain transfer function that filters the original signal to be measured.

For an LTI system with impulse response *h*(*t*), the output recorded *s*(*t*) is related to the entry *e*(*t*) by relation 2:

(2) $$s(t)=e(t)*h(t)=\int_{-\infty }^{+\infty }e(\tau ).h(t-\tau )d\tau$$

where * is the convolution operation.

In the frequency domain, relation 1 gives Formula (3):

(3) S(f)=E(f).H(f)

**Figure 8:**
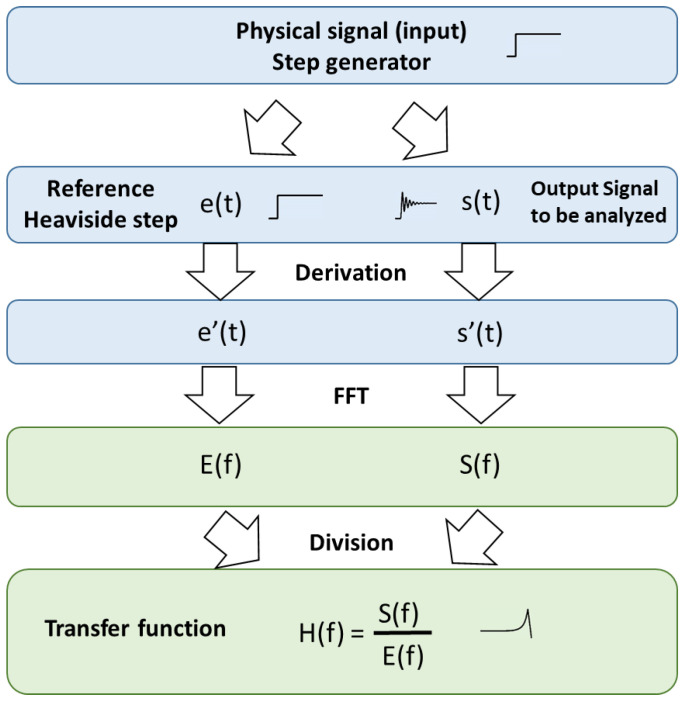
Process leading to the transfer function determination of a measurement chain.

We consider the pressure step generated by the shock tube as the input signal, used to excite the transducers. The process to obtain the transfer function of the measurement chain is detailed in Figure 8.

In the method presented in this paper, we don’t consider using a pressure reference sensor to determine the transfer function of the measurement chain to be tested, since its transfer function is not well-known because of the lack of a primary standard for dynamic pressure calibration. An error would be made when considering its Fourier transform as a Heaviside function, resulting in the deterioration of the transfer function determination of the transducer and measurement chain to be characterized. We do not consider either the determination of the reference input by using the analytical Rankine–Hugoniot model describing the pressure step generation in the shock tube based on initial pressure and temperature conditions in both chambers and the Mach number measurement within the driven section. Indeed, the two PCB Piezotronics 137B26 sensors positioned on the driven chamber are not sufficiently accurate regarding response time, to give a reliable chronometry determination; they are only considered to give qualitative information.

We thus choose arbitrarily to approximate the input pressure step, as an ideal Heaviside function, whose amplitude is given by the sensor to be characterized itself: the plateau pressure value is determined by the mean of the pressure plateau in the zone where its amplitude doesn’t exceed 3 times the RMS of the step. The rise-time of the Heaviside step is positioned arbitrarily at the beginning of the step pressure measurement of the sensor. Manually adjusting the reference step on the signal lead to the loss of the phase information, degrading the response time of the transducer. This limitation is accepted in this study.

**Figure 9:**
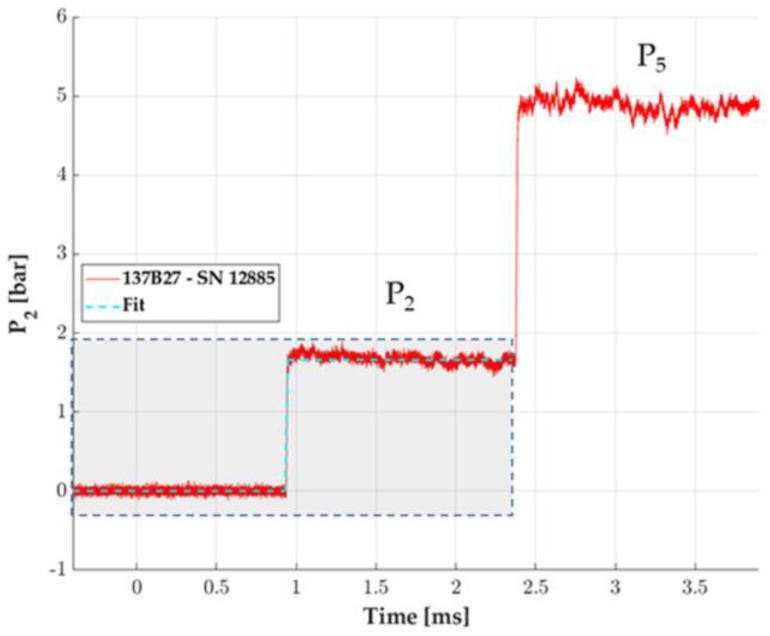
Example of pressure steps measured by a 137B27 pencil probe in the shock tube: P_2_ for incident shock, P_5_ for reflection shock. The zone of interest is highlighted by the dashed zone: P_2_ (red) and reference Heaviside step estimation (dot line in light blue), which will be the reference for the transfer function calculation in incident configuration.

We assume in our method a supposed ideal shock tube that generates a pressure step considered as a reference from Rankine–Hugoniot theory visible in light blue in Figure 9, based on the dashed zone of the pressure signal corresponding to the incident state P_2_. The transition time and amplitude of this artificial Heaviside signal, used for the transfer function determination on incident configuration, are determined concerning the red P_2_ signal corresponding to the experimental 137B27 pressure measurement.

Generating a fast Fourier transform (FFT) in the time domain confers better linearity to the bandwidth. Still, since we aim to focus on the cut-off frequency observation, we generate a step directly in the frequency domain to remove aliasing. It can be useful, when the measured signal is noisy, to oversample the signal using the zero-padding technique, and then apply a low pass filter.

Performing a FFT on a signal without degrading the information, requires a finite signal. A useful property of the Fourier transform is considered in our methodology: differentiating a function concerning time yields only the constant multiple of the initial function in the frequency domain, as presented in relation (4):

(4) $$\frac{d}{dt}x(t)↔j\omega X(\omega )$$

The step-by-step procedure leading to the transfer function determination of the measurement chain, especially the sensor response, is detailed.

Regarding the signal obtained by the sensor to be characterized submitted to a step pressure input:

1. Select the zone of interest for the temporal signal of the measurement chain.
2. Derivate the signal to obtain a finite signal and remove the offset, without deteriorating the information, as presented in Formula (3).
3. Calculate the FFT of the signal.

For the reference signal that will be approximated as a Heaviside step:

1. Select the zone of interest for the signal used as a reference sensor or that will be approximated as an ideal step (P_2_ for incident solicitation).
2. Determine the region of interest of plateau high and low to average the value and create a Heaviside reference input (respecting the Shannon theorem).
3. Determine the instant of the transition manually.
4. Derivate the signal to remove the offset on the signal, without deteriorating the information, as presented in Formula (3).
5. Calculate the FFT of the reference signal.

The transfer function is given by the ratio between the FFT of the signal measured by the sensor to be characterized and the FFT of the Heaviside reference step estimated to be generated by an ideal shock tube.

This method allows the comparison in gain of the transfer function of the commercial sensors, or of new prototypes characterized on a laboratory shock tube. Section 3 exposes the benefits of analyzing the transfer function of such probes. The frequency response function is generally expected in both gain and phase. The limitation of our method is the degradation of the phase information since the signals are temporally shifted. Note that the phase can also have an impact on the distortion of the input signal (in its temporal form), but this effect is not considered.

## 3. Results

### 3.1. Benefits of Using an Optimized Pencil Probe: Comparison with Commercial Sensors in Laboratory and Blast Experiments

The transfer function gives a better understanding of the sensor behavior in the frequency domain. Manufacturers often estimate the natural frequency, but it is difficult to precisely know the range of frequency where it is possible to use the sensor without degrading the measurement.

**Figure 10:**
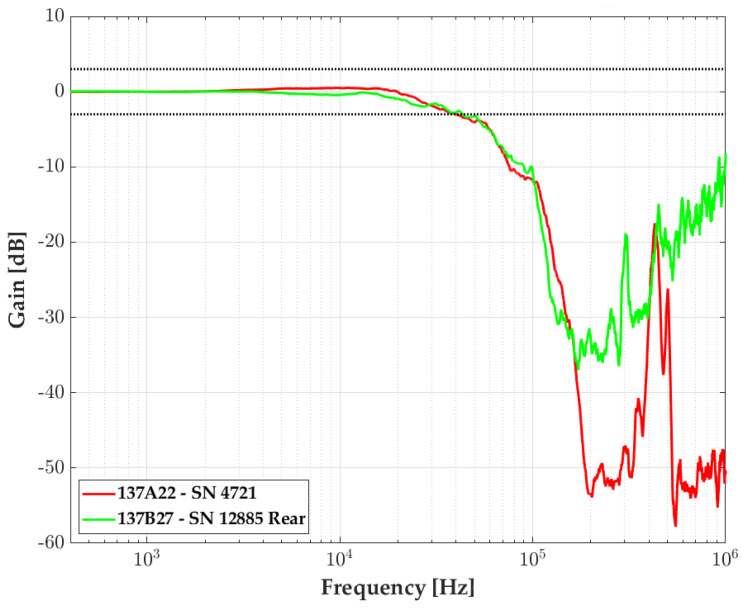
Transfer function comparison for piezoelectric probe sensors: 137A22 (red), 137B27 rear (green) for a pressure step of 15 bar. The ± 3dB zone is delimited by horizontal black-dot lines.

Thanks to shock tube experiments, we can compare several manufacturer transducer responses. In Figure 10, we can see the transfer functions of two PCB Piezotronics 35-bar probes: one belonging to the 137A22 model (red), with a single piezoelectric transducer, and the second belonging to the 137B27 model composed of two piezoelectric transducers, only the rear one (in green) is presented. Both present a flat band at ±3 dB, delimited by the two horizontal black-dot lines, up to 30 kHz, consistent with the rule of thumb mentioned previously. The two sensing elements from the 137B and 137A probe family show good agreement.

This procedure is well suited to compare our optimized custom sensor with commercial ones.

**Figure 11:**
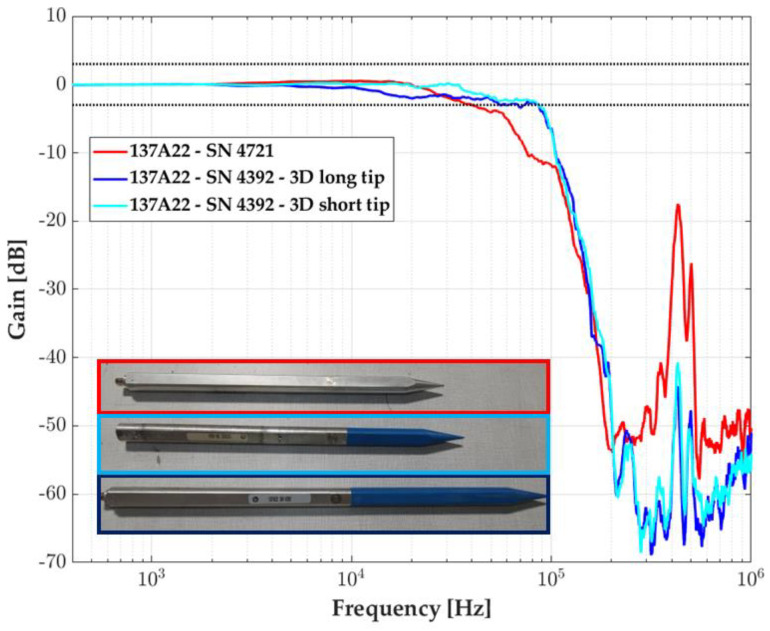
Transfer function of the pencil probe 137A22: the commercial one (red) and two pencils with modified tips, short (light blue), and long (dark blue). The ±3dB zone is delimited by horizontal black dot lines.

Figure 11 shows the response of the optimized probe presented in Section 2 in blue shades, light for the short plastic tip and dark for the long one. The bandwidth at −3 dB is three times that of another 137A22 commercial probe (in red): 90 kHz instead of 30 kHz. The resonant frequency is visible within the range given by the datasheet manufacturer, approximately 440 kHz. The pencil modification does not affect the transducer resonant frequency value, it only attenuates the gain.

The difference in length of the plastic tip of 10.2 cm between the short and the long tip, appears to have no influence on the bandwidth since the frequency at −3 dB is identical, around 85.5 kHz. In the free-field experiments, we only focus on the 137A22 pencil probe mounted with a 3D-printed long tip.

**Figure 12:**
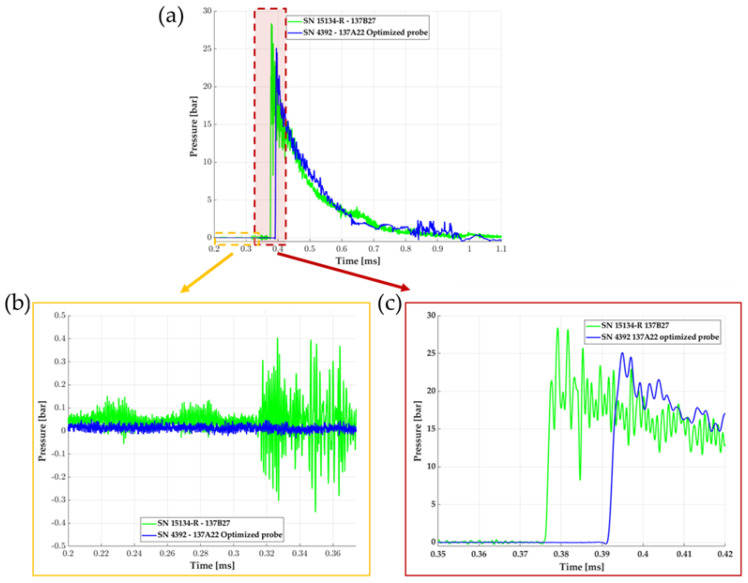
(**a**) Pressure measurement of two probes with 2.459 kg of composition 1, located at the same distance from the center of the charge (~1 m): one commercial 137B27 (green) and the plastic-tip custom one 137A22 (blue). (**b**) Zoom on the signal noise before the arrival of the shock wave (yellow dashed zone visible in (**a**)). (**c**) Zoom on the pressure rise region (red dashed zone visible in (**a**)).

To confirm the bandwidth improvement of the optimized custom probe in the experimental field, we compared the measurement of two sensors placed at the same distance from the charge (~1 m), but with a different reference sensor: a 137B27 commercial probe considering its rear transducer (green) and a 137A22 optimized plastic tip (blue). Figure 12a illustrates the pressure history recorded from those pressure transducers. The pressure wave decreases in intensity with probe distance from the charge, but the overpressure front conserves its very short rise time, in the range of tens of nanoseconds, corresponding to the thickness of a few mean free path molecules at the front [22]. It can be observed that significant perturbations (i.e., pressure oscillations) are present along the record coupled with a large overshoot which represents an exaggerated peak overpressure with the commercial probe, which is a common issue with blast measurements [23,24].

Figure 12 is an overview highlighting two beneficial effects of the plastic tip optimization; the dashed zones, respectively red and yellow, correspond to focus images in the next subfigures:

- in Figure 12b, we observe parasites on the green measurement from the commercial probe visible at the step of the signal, before the arrival of the shock wave on the transducer (for times < 0.4 ms). It is not visible on the probe with the optimized plastic tip. The RMS values of the signals were calculated before the arrival of the shock wave and presented in Table 6: the disappearance of those parasites validates the role of the tapered plastic tip in the attenuation of the acoustic wave propagation.
- Figure 12c focuses on the pressure rise region (red dashed zone). A resonant frequency is more present in the pressure measurement of the commercial probe. The improvement of the two-material custom probe here is validated: it reduces the influence of the acoustic waves within the probe and limits the resonant peak of the transducer. In Table 7, we detail the attenuation ratio of the resonant frequency gain (at 440 kHz) for the four combinations of composition and high explosive weights: the resonant frequency reduction average is approximately 66%. Note that the arrival time detection of the shock wave is smaller (0.363 ms) for the commercial probe, than for the optimized one (0.378 ms): this difference of 15 µs is due to the distance from the charge between the two sensors (2 cm, as presented in Table 4), coherent with an estimated velocity of the shock wave of 1300 m/s.

**Figure 13:**
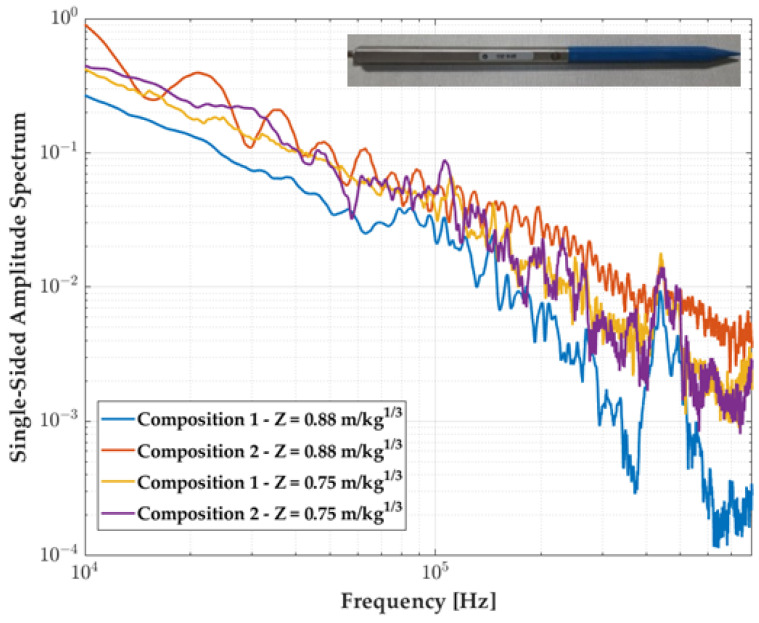
Experimental pressure measurements in the frequency domain for the 137A22 probe with the optimized tip. The colors correspond to the four experiments performed, characterized by the Z factor.

The behavior in terms of the frequency response of such an optimized probe was evaluated by the four experiments of the campaign. We see in Figure 13 the Fourier transform of the signal: a resonance peak around 440 kHz is visible on all signals, the spectrum being similar through the composition weight, the optimized design has no effect on the frequency response of the measurement chain.

### 3.2. Deconvolution Process

**Figure 14:**
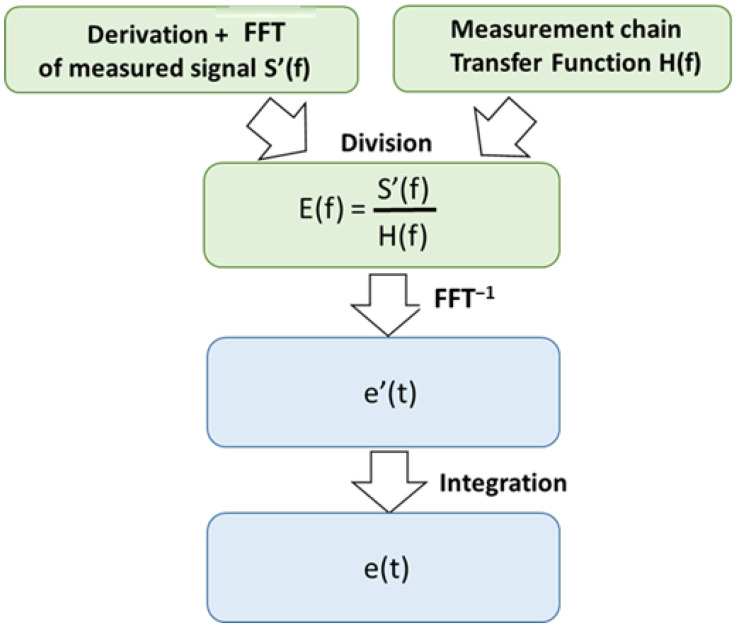
Process leading to the deconvolution of an experimental signal with the transfer function of a previously determined measurement chain.

This work is based on several papers on deconvolution filters for analyzing dynamic measurement processes [25,26,27,28]. The method presented in Figure 14 shows the deconvolution results in a division in the frequency domain of the experimental signal FFT by the transfer function of the sensor. Using the FFT^−1^ and integrating the signal results in an experimental signal corrected for gain defects of the characterized measurement chain (mostly the sensor).

**Figure 15:**
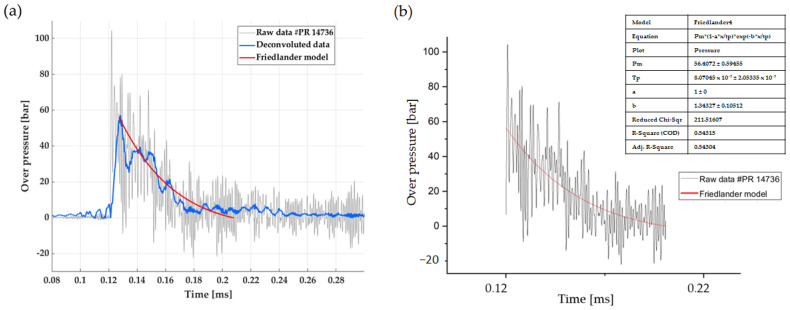
Experimental pressure-time profile obtained from near-field experimentation: (**a**) raw measurement (grey) of a 137B28 probe sensor located at 0.55 m from the center of the charge (Z = 0.41 m.kg^−1/3^), and its deconvolution (in gain only) in blue. The red curve estimates the Friedlander fit of the pressure decay to determine the overpressure peak. (**b**) Details of the Friedlander fit (red) presented in (**a**) are deduced from the raw data (grey).

This deconvolution method makes it possible to “subtract” the imperfections (related to the measurement chain associated with the sensor) from the measurement signal, giving a result more closely resembling the physical phenomenon. An example in Figure 15 is given where the 137B28 pressure signal is parasitized by the resonant frequency excitation of the transducer in a near-field blast experiment.

We consider a 137B28 pencil probe positioned at 0.55 m from the center of the charge, for 2.357 kg of composition 1.

As mentioned in Table 4, this reference presents the same features as the 137B27 probe regarding bandwidth and rise time; it differs only by the measurement range, which is twofold higher: 70 bars. The pressure signal is visible in grey in Figure 15: it shows significant noise due to the probe’s location inside the fireball expansion after the explosive ignition. One can also see the oscillations due to the resonant frequency that degrades the maximum overpressure peak determination.

The inverse transfer function (based on the transfer function determined in the incident configuration on the shock tube) is used to reconstruct the probe signal in the time domain, as visible in the light blue signal. As mentioned, since we do not consider phase deconvolution in our method, we applied a temporal shift to superimpose the two signals. Despite a slightly degraded rise-front, this signal appears to filter quite well the raw signal.

To estimate the maximum peak overpressure from the raw signal, we can apply a method consisting in determining this value as the intersection between the rise-front of the signal and the extrapolation of a Friedlander-type signal. Such a pressure-time profile is usually modeled by this kind of function [29,30]. The positive phase of the free-field air blast wave can be described by the ‘modified Friedlander equation’ [31]. Given Formula (5), the pressure *P* at time *t* is:

(5) $$P(t)=P_{0}+P_{max}\left(1-\frac{\left(t-t_{a}\right)}{t_{d}}\right)e^{-b\frac{\left(t-t_{a}\right)}{t_{d}}}$$

where, *t* is measured from the arrival time *t_a_*, *t_d_* is the time interval called positive duration, *P_0_* is the reference pressure (in most cases the atmospheric pressure), *b* is known as the waveform parameter that controls the decay of the pressure-time curve, and *P_max_* is the maximum pressure to be determined (peak-overpressure).

**Figure 16:**
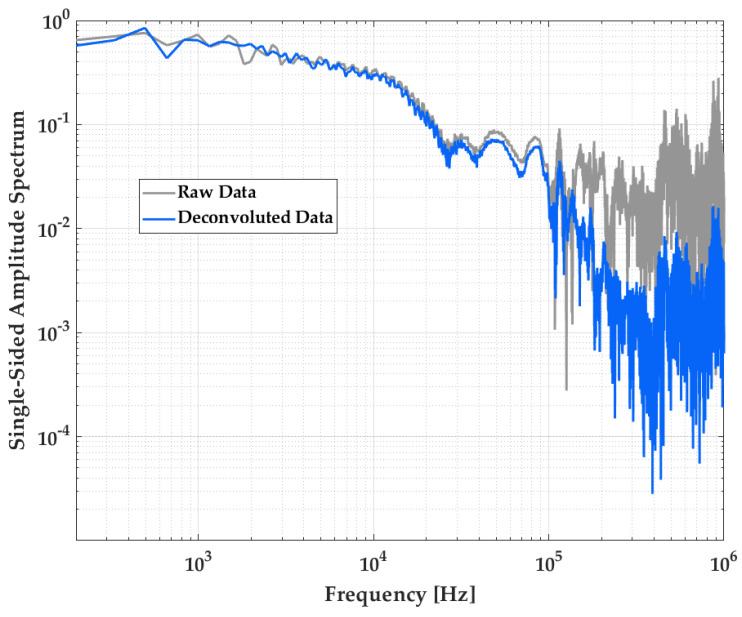
Spectra of the pressure measurement presented in Figure 15: raw measurement of the 137B28 probe sensor (grey), and deconvoluted measurement (blue).

This model gives a value of (56.41 ± 0.59) bar, where the deconvoluted signal gives an overpressure peak at 57.34 bar. We can see a good correlation between the Friedlander fit from the raw signal and the deconvoluted signal, regarding maximum peak determination. Moreover, Figure 16 compares the Fourier transform of the deconvoluted signal (in blue) to the raw one (in grey): the filtering function in frequencies above 100 kHz given by the deconvolution process is clear.

## 4. Discussion and Perspectives

In this paper, two methods are shown to improve the pressure measurements of blast waves in free-field experiments, especially considering the near-field zone, for scaled distances Z < 0.4 m.kg^−1/3^. The aim is to eliminate signal distortions coming either from mechanical limitations or from the transfer function of the measurement chain itself.

Firstly, a new kind of probe is presented: based on modifying the tip from a commercial piezoelectric pencil probe, it shows a larger bandwidth to measure the shock wave overpressure in real time. The material replacement of the tip at the front of the pencil does not impact the transducer integration within the body, which is still the manufacturer’s work. Hence, a modified probe and a commercial one present the same transducer as well as the same transducer mounting within the pencil. The performance differences observed between them in terms of bandwidth improvements are only due to a mechanical improvement leading to the limitation of an acoustic wave’s influence. In this study, no pencil cutting was performed to remove and change the tip. The replacement presented in this study is limited to a few commercial pencil probe families whose design allows a tip replacement (generally, the tip is fixed and maintained by a screw on the body of the pencil probe). The mounting of the custom plastic tip is more fragile than the metal one, the plastic often deteriorates near the mounting screw in the near-field zone, but it is easy to replace it when needed. The first experiments presented were conducted mostly at scaled distances between 0.5 m.kg^−1/3^ and 1 m.kg^−1/3^ to ensure conditions that facilitate the characterization of the optimized pencil probe without damaging it.

We show that the characterization, in the frequency domain, of a pressure measurement chain is of prime importance since it can facilitate the true impact evaluation of optimized devices relative to commercial ones. A better understanding of the useful bandwidth is an important parameter since Chalnot et al. have shown its importance in estimating the overpressure magnitude at the blast wave front, to obtain the desired accuracy [32].

The mechanical filtering improvements obtained by modifying a pressure commercial probe 137A22 were tested, and a noise reduction in the pressure measurement was found. Ongoing studies are devoted to material evaluation to compare different kinds of tips, as well as different designs for each given material. The laboratory shock tube, which offers defined pressure steps, is particularly useful to help characterize the custom pencil probe transfer function, for the impact evaluation of each parameter of interest. Simulations will be performed to confirm the behaviors observed. New experiments will be conducted in a free field with optimized pencil probes at lower scaled distances to see the true improvement to the pressure measurements near the charge, where the parasites and ringing oscillations are the highest.

Secondly, the deconvolution process is a method for improving experimental results by filtering transient measurements without deteriorating the frequency domain of interest. It can be of real interest for low-scaled distances. This paper presents a draft method that will be developed in the future. These preliminary results show a major drawback that needs to be corrected in order to obtain a robust method: it is important to consider not only the gain of the transfer function but also its phase. This lack of information prevents determination of the arrival time of a shock wave, or the response time of a dynamic system after deconvolution. A real comparison of maximum overpressure peak estimation and impulsion between deconvoluted signals and the Friedlander fit model must be performed to show the true benefit of such a methodology. Moreover, the reconstruction of the experimental signal by the deconvolution of the measurement chain transfer function must be associated with uncertainty based on a reliable evaluation of the pressure sensitivity evolution [33,34,35].

Using a shock tube to accurately determine the transfer function of a pressure measurement chain is key. The generation and the characterization of the reference step pressure in a laboratory shock tube to determine the transfer function of the measurement chain need to be improved. Work is ongoing to obtain a reference input by using the analytical Rankine–Hugoniot model describing the pressure step generation in the shock tube based on initial pressure and temperature conditions in both chambers and the Mach number measurement within the driven section with piezoelectric pins [36].

## 5. Conclusions

We summarize here the main conclusions, bearing in mind that the studies presented in this paper are following the pressure measurement improvements for experimental blast wave characterizations, for scaled distances where the commercial pencil probes are unable to give useful measurements. The full characterization of the transfer function of the measurement chain with a shock tube is an asset to better evaluate its performance. We present in this study the first results of transfer function determination regarding commercial pencil probes and optimized ones. Dynamic errors occur whenever the bandwidth of the employed measurement chain is small compared to the input signal’s bandwidth. In our case, two approaches are followed to reduce these errors. Firstly, this study highlights the benefits of the use of a two-material pencil probe that gives improvements in terms of useful bandwidth frequency. Next, a signal processing method to calibrate the dynamic response of the measurement chain in the frequency domain is investigated: a deconvolution filter, based on a reliable characterization of the measurement chain transfer function, can improve pressure measurement.

This paper has demonstrated that basic research and development work in both the mechanical optimization and signal processing fields have been carried out and will continue to answer the need for reliable pressure measurement in the free-field environments, for small-scaled distances, reaching values below 0.4 m.kg^−1/3^.

## Figures and Tables

**Table 1:** Pencil probes were characterized in this study. The optimized pencil probe corresponds to the commercial probe with the modified tip.

	Reference	Serial Number	Pencil Probe	Tip	Distance Tip-Sensor [mm]	Resonant Frequency
**1 sensing** **element**	137A22	4392	Optimized	Plastic	259	≥500 kHz
	137A22	4392	Optimized	Plastic	157	≥500 kHz
	137A22	4721	Commercial	Metal	157	≥500 kHz
**2 sensing** **elements**	137B27	15134-R	Commercial	Metal	259 (from rear sensing element)	≥400 kHz
	137B28	14736-R	Commercial	Metal	259 (from rear element)	≥400 kHz
	137B28	14736-F	Commercial	Metal	157 (from front sensing element)	≥400 kHz

**Table 2:** Cable types and lengths are used in both free-field and laboratory studies. For the free-field configuration, the long coaxial cables used to transfer the signal to the acquisition system, located less than a hundred meters from the explosive source, are buried to protect them from the blast effects.

		137A22 4392 Long Tip	137A22 4392 Short Tip	137A22 4721	137B27 15134	137B28 14736
Free-field experiments	Low-noise cable before conditioner [m]	4	4	4	4	4
	KX15 cable before conditioner [m]	15	15	15	15	15
	KX15 cable after conditioner [m]	54	54	54	54	54
Laboratory	Low-noise cable before conditioner [m]	3	3	3	3	-
	KX15 cable after conditioner [m]	20	20	20	20	-

**Table 3:** Compositions of high explosives used in these free-field detonation experiments: the HMX/binder weight ratio varies between the two compositions. Two masses are studied for each composition.

Reference	HMX/Binder Weight Ratio	Low Mass [kg]	High Mass [kg]
Composition 1	88%/12%	1.639	2.459
Composition 2	86%/14%	1.643	2.466

**Table 4:** Piezoelectric pencil probes were used in these free-field experiments.

Reference	Pencil Probe Configuration	Serial Number	Distance from Charge Center [m]	Color in Graphs	Measurement Range [bar]
137B28 Front	Commercial	14736-F	0.45	Grey	70
137B28 Rear	Commercial	14736-R	0.55	Black	70
137B27 Rear	Commercial	15134-R	1.01	Green	35
137A22	Optimized	4392	1.03	Blue	35
137A22	Commercial	4721	1.29	Red	35

**Table 5:** Scaled distance calculation for compositions 1 and 2, whose pressure signals are presented in this paper. Both compositions of the same weight have a comparable Z value.

Reference	Z for Comp. 1 of 1.639 kg and Comp. 2 of 1.643 kg [m.kg^−1/3^]	Z for Comp. 1 of 2.459 kg and Comp. 2 of 2.466 kg [m.kg^−1/3^]
137B28 Front	0.38	0.33
137B28 Rear	0.46	0.41
137B27 Rear	0.86	0.75
137A22 optimized	0.88	0.77
137A22	1.09	0.95

**Table 6:** RMS measurement of the pressure signal before shock wave arrival for the optimized 137A22 pencil probe used in this study and comparison with the commercial 137B27 probe.

Composition–Mass	Commercial Probe RMS [bar]	Optimized Probe RMS [bar]
1–1.639 kg	0.05	0.01
2–1.643 kg	0.07	0.02
1–2.459 kg	0.08	0.01
2–2.466 kg	0.27	0.01

**Table 7:** Resonant frequency gain attenuation between the 137B27 commercial probe and the custom plastic-tip probe, for the four combinations of composition and mass of the high explosive (HE) samples.

HE Composition	HE Weight [kg]	Resonant Frequency Attenuation Factor at 440 kHz
1	1.639 kg	42.9%
2	1.643 kg	72.7%
1	2.459 kg	75.0%
2	2.466 kg	73.7%

## Data Availability

Not applicable.

## References

[1] Walter P.L. (2004). Air-blast and the science of dynamic pressure measurements. Sound Vib..

[2] Lukić S., Draganić H., Gazić G., Radić I. Statistical analysis of blast wave decay coefficient and maximum pressure based on experimental results. Proceedings of the RISK/SAFE 2022.

[3] Silver P.L. (2006). Blast Overpressure Measurement for CFD Model Validation in the Development of Large Caliber Gun Systems.

[4] Showalter B. (2020). Utilization of Pencil Probes in Blast Experiments for the Explosive Effects Branch.

[5] Dvořák P., Hejmal Z., Dubec B., Holub J. Use of Pencil Probes for Blast Pressure Measurement. Proceedings of the 2021 International Conference on Military Technologies (ICMT).

[6] Skriudalen S., Skjold A., Hugsted B., Teland J.A., Huseby M. (2014). Misalignment Effects Using Blast Pencil Probes.

[7] Draganić H., Varevac D., Lukić S. (2018). An Overview of Methods for Blast Load Testing and Devices for Pressure Measurement. Adv. Civ. Eng..

[8] Kingery C.N., Bulmash G. (1984). Airblast Parameters from TNT Spherical Air Burst and Hemispherical Surface Burst.

[9] Shin J., Whittaker A.S., Cormie D. (2015). Incident and Normally Reflected Overpressure and Impulse for Detonations of Spherical High Explosives in Free Air. J. Struct. Eng..

[10] Karlos V., Larcher M., Solomos G., European Commission Joint Research Centre (2016). Analysis of Blast Parameters in the Near-Field for Spherical Free-Air Explosions.

[11] Bean V.E. (1994). Dynamic Pressure Metrology. Metrologia.

[12] Downes S., Knott A., Robinson I. (2014). Towards a shock tube method for the dynamic calibration of pressure sensors. Philos. Trans. R. Soc. Lond. A Math. Phys. Eng. Sci..

[13] Persico G., Gaetani P., Guardone A. (2005). Dynamic calibration of fast-response probes in low-pressure shock tubes. Meas. Sci. Technol..

[14] Zahnd A., Lorenzo R., Clausen M., Hulliger B., Seitz A. Shock tube verification of the factory calibration of pencil probes. Proceedings of the MABS 24.

[15] Piezotronics P. https://www.pcb.com/.

[16] Klaus L., Bruns T., Volkers H. (2015). Calibration of bridge-, charge- and voltage amplifiers for dynamic measurement applications. Metrologia.

[17] ENDEVCO (1997). Piezoresistive Pressure Transducters Insutruction Manual.

[18] Guerke G.H. Evaluation of blast pressure measurements. Proceedings of the 12th International Symposium Military Aspects of Blast and Shock (MABS).

[19] Lavayssière M., Luc J., Lefrançois A. Experimental Studies Around Shock Tube for Dynamic Calibrations of High-Frequency Pressure Transducers. Proceedings of the 31st International Symposium on Shock Waves 1.

[20] Duff R.E. (1959). Shock-Tube Performance at Low Initial Pressure. Phys. Fluids.

[21] Hjelmgren J. (2003). Dynamic Measurement of Pressure: A Literature Survey.

[22] Sarraf C., Damion J.-P. (2018). Dynamic pressure sensitivity determination with Mach number method. Meas. Sci. Technol..

[23] Kinney G. (1968). Engineering Elements of Explosions.

[24] Skotak M., Alay E., Chandra N. (2018). On the Accurate Determination of Shock Wave Time-Pressure Profile in the Experimental Models of Blast-Induced Neurotrauma. Front. Neurol..

[25] Eichstädt S., Elster C., Esward T.J., Hessling J.P. (2010). Deconvolution filters for the analysis of dynamic measurement processes: A tutorial. Metrologia.

[26] Elster C., Link A. (2008). Uncertainty evaluation for dynamic measurements modelled by a linear time-invariant system. Metrologia.

[27] Eichstadt S., Wilkens V., Dienstfrey A., Hale P., Hughes B., Jarvis C. (2016). On challenges in the uncertainty evaluation for time-dependent measurements. Metrologia.

[28] Matthews C., Pennecchi F., Eichstädt S., Malengo A., Esward T., Smith I., Elster C., Knott A., Arrhén F., Lakka A. (2014). Mathematical modelling to support traceable dynamic calibration of pressure sensors. Metrologia.

[29] Ismail M.M., Murray S.G. (1993). Study of the Blast Wave Parameters from Small Scale Explosions. Propellants Explos. Pyrotech..

[30] Rigby S., Tyas A., Fay S., Clarke S., Warren J. Validation of semi-empirical blast pressure predictions for far field explosions—Is there inherent variability in blast wave parameters?. Proceedings of the 6th International Conference on Protection of Structures against Hazards.

[31] Baker W.E. (1973). Explosions in Air.

[32] Chalnot M., Pons P., Aubert H. (2022). Frequency Bandwidth of Pressure Sensors Dedicated to Blast Experiments. Sensors.

[33] Eichstadt S., Makarava N., Elster C. (2016). On the evaluation of uncertainties for state estimation with the Kalman filter. Meas. Sci. Technol..

[34] Amer E., Jönsson G., Arrhén F. (2022). Towards traceable dynamic pressure calibration using a shock tube with an optical probe for accurate phase determination. Metrologia.

[35] Yao Z., Li Y., Ding Y., Wang C., Yao L., Song J. (2022). Improved shock tube method for dynamic calibration of the sensitivity characteristic of piezoresistive pressure sensors. Measurement.

[36] Sarraf C., Damion J.P. (2021). Method for Dynamic Calibration of Pressure Sensors in Gazeous Media.

